# Characterization and genome functional analysis of a novel metamitron-degrading strain *Rhodococcus* sp. MET via both triazinone and phenyl rings cleavage

**DOI:** 10.1038/srep32339

**Published:** 2016-08-31

**Authors:** Hua Fang, Tianheng Xu, Duantao Cao, Longyin Cheng, Yunlong Yu

**Affiliations:** 1 Institute of Pesticide and Environmental Toxicology, College of Agricultural and Biotechnology, Zhejiang University, Hangzhou 310058, China

## Abstract

A novel bacterium capable of utilizing metamitron as the sole source of carbon and energy was isolated from contaminated soil and identified as *Rhodococcus* sp. MET based on its morphological characteristics, BIOLOG GP2 microplate profile, and 16S rDNA phylogeny. Genome sequencing and functional annotation of the isolate MET showed a 6,340,880 bp genome with a 62.47% GC content and 5,987 protein-coding genes. In total, 5,907 genes were annotated with the COG, GO, KEGG, Pfam, Swiss-Prot, TrEMBL, and nr databases. The degradation rate of metamitron by the isolate MET obviously increased with increasing substrate concentrations from 1 to 10 mg/l and subsequently decreased at 100 mg/l. The optimal pH and temperature for metamitron biodegradation were 7.0 and 20–30 °C, respectively. Based on genome annotation of the metamitron degradation genes and the metabolites detected by HPLC-MS/MS, the following metamitron biodegradation pathways were proposed: 1) Metamitron was transformed into 2-(3-hydrazinyl-2-ethyl)-hydrazono-2-phenylacetic acid by triazinone ring cleavage and further mineralization; 2) Metamitron was converted into 3-methyl-4-amino-6(2-hydroxy-muconic acid)-1,2,4-triazine-5(4H)-one by phenyl ring cleavage and further mineralization. The coexistence of diverse mineralization pathways indicates that our isolate may effectively bioremediate triazinone herbicide-contaminated soils.

Metamitron (4-amino-3-methyl-6-phenyl-1,2,4-triazin-5(4H)-one) is a selective systemic triazinone herbicide used as pre- and/or post-emergence in sugar beet, onion, and bean crops to control turf grass and broadleaf weeds^1^. It acts as an inhibitor of photosystem II and induces chlorotic and necrotic symptoms in leaves^2^. The recommended doses of metamitron are 1.4–2.0 kg/ha and 0.35–0.70 kg/ha for pre-emergence and post-emergence applications, respectively^3^. Some researchers reported that the degradation half-life of metamitron in soil ranged from less than 5 weeks to more than 14 weeks depending on the soil type and environmental conditions, such as temperature and moisture^2^,^4^. Due to its chemical stability and mobility, metamitron residues are often found in soil, plants, surface water, and groundwater^1^,^5^ and may pose a potential risk to the environment^6^. Therefore, there is an increasing concern regarding the rapid degradation or detoxification of metamitron residues in the environment.

Microbial degradation plays a major role in the removal of metamitron and other triazinone herbicides in the environment^7^,^8^,^9^,^10^. This finding was in agreement with the notable degradation of metamitron in soils that received successive treatments with this herbicide compared to control soil^11^. However, only few strains, capable of degrading metamitron, have been documented including *Arthrobacter* sp. DSM 20389 and *Rhodococcus* sp. 0246b. These strains metabolize metamitron through the separate cleavage of the triazinone ring or the phenyl ring^12^. At present, bacterial whole genome sequencing and functional annotation is a promising approach to identify degradation genes and elucidate their roles in degradation pathways through high-throughput sequencing. To the best of our knowledge, the biodegradation mechanism of metamitron has not been comprehensively explored using integrated genomic and high performance liquid chromatography-tandem mass spectrometry (HPLC-MS/MS) analyses.

In the present study, a novel bacterial strain (*Rhodococcus* sp. MET) capable of utilizing metamitron as the sole carbon and energy source was isolated and its degradation capability was explored. The objectives of this study were as follows: 1) to measure the morphological, physiological and biochemical characteristics of the isolate *Rhodococcus* sp. MET; 2) to conduct genome sequencing of *Rhodococcus* sp. MET and to determine its functional annotation; 3) to examine the effect of the initial substrate concentration, pH and temperature on the degradation of metamitron by the isolate *Rhodococcus* sp. MET; and 4) to reveal the potential biodegradation pathway of metamitron based on functional annotation of its genome and HPLC-MS/MS analysis. This study will provide new insights into the biodegradation mechanism of triazinone herbicides.

## Results and Discussion

### Recovery evaluation

To confirm the validity of the metamitron extraction method, recovery studies were performed at three spiking levels of 1, 10, and 100 mg/l in 20 ml of sterile MSM. The fortified recoveries of metamitron at levels of 1, 10, and 100 mg/l in MSM were 91.3%, 89.1%, and 89.2% with relative standard deviations of 4.9%, 4.2%, and 1.3%, respectively. The limit of detection (LOD) and the limit of quantitation (LOQ) of metamitron by HPLC were 0.1 mg/l. These data indicate that the extraction method is satisfactory for the analysis of residual metamitron.

### Characterization of the isolate MET

**Figure 1:**
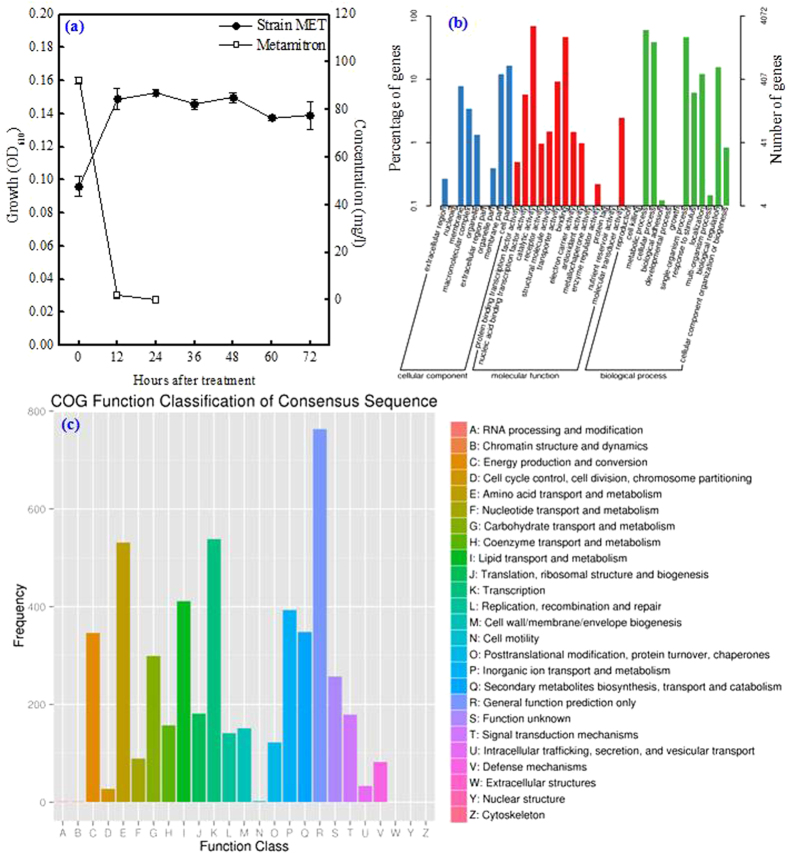
Growth of the isolate MET in mineral salts medium supplemented with 100 mg/l of metamitron at pH 7.0 and 30 °C (**a**), genome function annotation of the isolate MET against the Gene Ontology (GO) database (**b**), and genome function classification of the isolate MET against the Clusters of Orthologous Groups (COG) database (**c**).

A bacterial strain capable of utilizing metamitron as the sole carbon and energy source was isolated from activated sludge and designated as MET. The growth curve of the isolate MET in sterile MSM supplemented with 100.0 mg/l of metamitron at pH 7.0 and 30 °C is shown in Fig. 1a. The rod-shaped cells were atrichous and asporous with a size of 0.7–1.0 μm × 1.2–2.5 μm. The initial colony was circular and transparent, with neat edge and uplift surface; the mature colony was light yellow, smooth and moist when grown on the LB plate. The isolate MET was Gram-positive. Its optimal growth pH and temperature were 7.0 and 30 °C, respectively. The relative utilization capacities of 95 different substrates by the isolate MET in the BIOLOG GP2 microplate are given in Table S1. The isolate MET was most closely related to genus *Rhodococcus* with 0.92 similarity (24 h) using the BIOLOG Microlog 4 database software. As shown in Table S2, the 16S rDNA sequence from the isolate MET had high similarity (99%) to members of genus *Rhodococcus*. Based to the above characteristics, the isolate was identified as *Rhodococcus* sp. MET.

### Genome properties of the isolate MET

The genome properties of the isolate MET are shown in Table 1. *Rhodococcus* is a metabolically versatile genus with remarkable ability to catabolize a wide range of organic compounds, such as organochlorine pesticides including hexachlorocyclohexanes and 1,1,1-trichloro-2,2-bis(4-chlorophenyl) ethane (*R.* strain IcdP1), nitrophenyl (*R. imtechensis* strain RKJ300), polychlorinated biphenyl (*R.* strain RHA1), alkane (*R. erythropolis* strain PR4) and hydrocarbons (*R. opacus* strain B4)^13^,^14^,^15^,^16^. The genome size of these five strains was 5.92–9.70 M, comprising 62.3–70.6% GC%, 6,043–8,816 genes, 5,009–8,465 proteins, 5–15 rRNA and 50–54 tRNA^13^,^14^,^15^,^16^. Genome functional annotation of the isolate MET against GO database is shown in Fig. 1b and Table S3. As shown in Fig. 1c, the functional classification of the isolate MET genome based on the COG database revealed 25 function classes including energy production and conversion, amino acid transport and metabolism, carbohydrate transport and metabolism, lipid transport and metabolism, and transcription. These sequence data can be used to predict genes for xenobiotic biodegradation and metabolism^16^. Integrated functional annotation of the isolate MET genome against the above seven databases found some dominant genes encoding diverse enzymes, including a hydrolase, deaminase, decarboxylase, and dehydrogenase, which could be responsible for different steps in the metamitron biodegradation process (Table S4). Similar to the isolate MET, stain RHA1 contained the genes encoding dehydrogenase, and stain RKJ300 harbored the genes encoding dehydrogenase, hydrolase and decarboxylase. Distinct to the isolate MET, strains RKJ300 and IcdP1 contained the genes encoding dioxygenase, which could initiate the oxidation of persistent organic pollutants^16^. Additionally, strain RKJ300 carried the genes encoding desulfurase, kinase, hydratase, monooxygenase, phosphatase, Co-A-transferase, hydroxylase, tautomerase, reductase and esterase, and strain RHA1 contained the genes encoding desulfurase, kinase, hydratase, oxygenase and ligase, which involved in the biodegradation of aromatic compounds^13^,^14^. Table S5 summarizes the rapid evolutionary gene detection of the isolate MET and its closely related strains IcdP1, RKJ300, RHA1, PR4 and B4, and their gene numbers with Ka/Ks ratio > 1 were 2, 2, 3, 0, 0 and 4. The collinearity analysis results on the protein sequences between the isolate MET and its closely related species *R. opacus* strain B4 are shown in Fig. S1. The Venn diagram shows 9, 76, 83, 1 and 0 genome-specific genes in the isolate MET and its closely related strains RHA1, PR4, B4 and ATCC 4277, respectively, and 3,566 shared genes in all these strains (Fig. S2).

### Effect of the initial metamitron concentration on biodegradation

**Figure 2:**
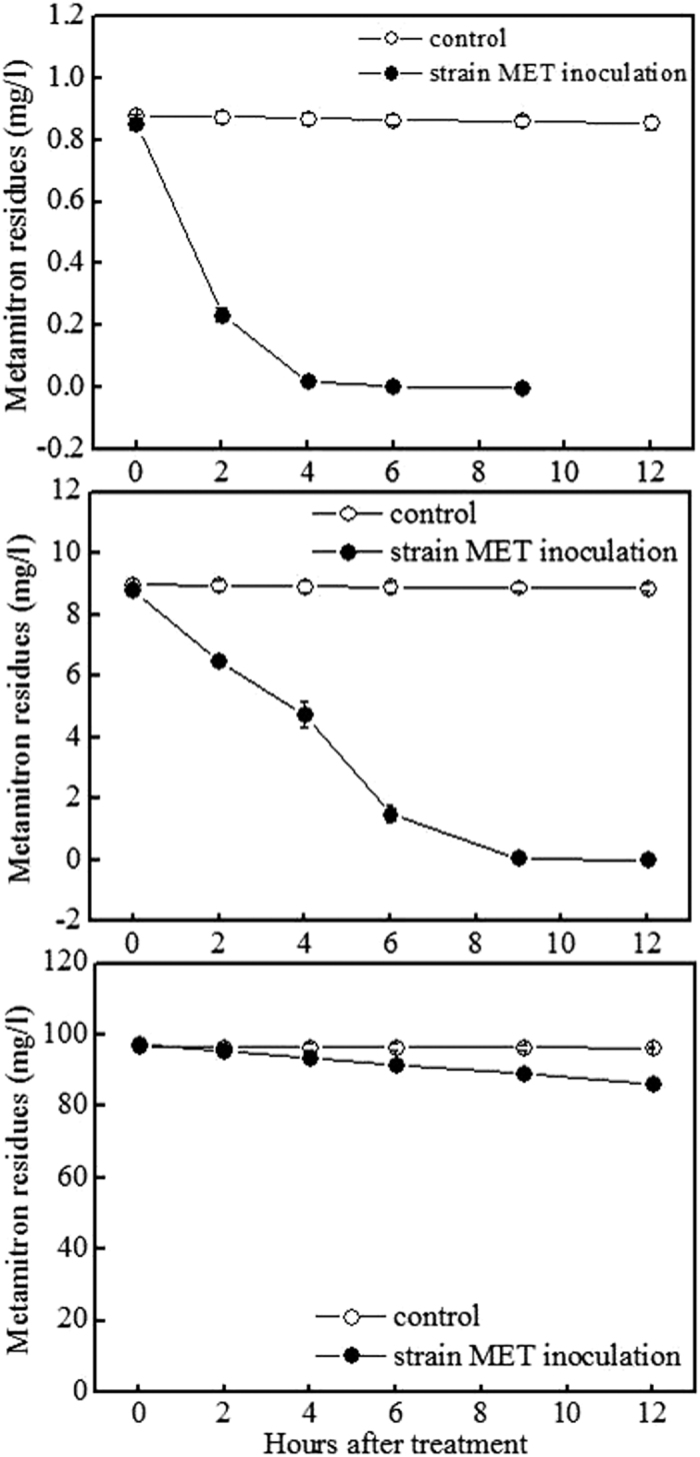
Degradation of metamitron at concentrations of 1, 10, and 100 mg/l by the isolate MET in mineral salts medium at pH 7.0 and 30 °C.

The effect of metamitron concentration on its biodegradation in MSM at pH 7.0 and 20 °C is shown in Fig. 2 and Table S6. As shown in Fig. 2, the degradation of metamitron by the isolate MET followed a pseudo first-order model. The dissipation rate of metamitron in MSM without inoculation of the isolate MET was less than 2.6%. No residual metamitron was detected in the liquid culture after 9 h of incubation with 1 mg/l of metamitron and 12 h of incubation with 10 mg/l of metamitron (Fig. 2). The degradation rates of metamitron at levels of 1, 10, and 100 mg/l by the isolate MET after 6 h of incubation were 0.14, 1.22, and 0.97 mg/l/h, respectively (Table 2). The results showed that the isolate MET could effectively degrade metamitron. Furthermore, the degradation rate obviously increased with the increased initial substrate concentration from 1 mg/l to 10 mg/l and subsequently decreased as the substrate concentration increased ranging from 10 mg/l to 100 mg/l. Similar to our results, the degradation of metamitron in soil followed first-order kinetics^4^. The degradation of the triazinone herbicide metribuzin (5–50 mg/l) by *Bacillus* sp. N1 in liquid medium also followed first-order kinetics, and the degradation rate decreased as the substrate concentrations increased from 50 mg/l to 100 mg/l^10^. Additionally, some researchers have reported that bacterial strains from the same genus *Rhodococcus* with the isolate MET used in this study could degrade other herbicides^17^,^18^. Xiong *et al*.^19^ isolated a bacterial strain *Rhodococcus* sp. BX2 that could degrade the herbicide bensulfuron-methyl, and the degradation rate increased with the increase in the substrate concentration.

### Effect of pH on metamitron biodegradation

**Figure 3:**
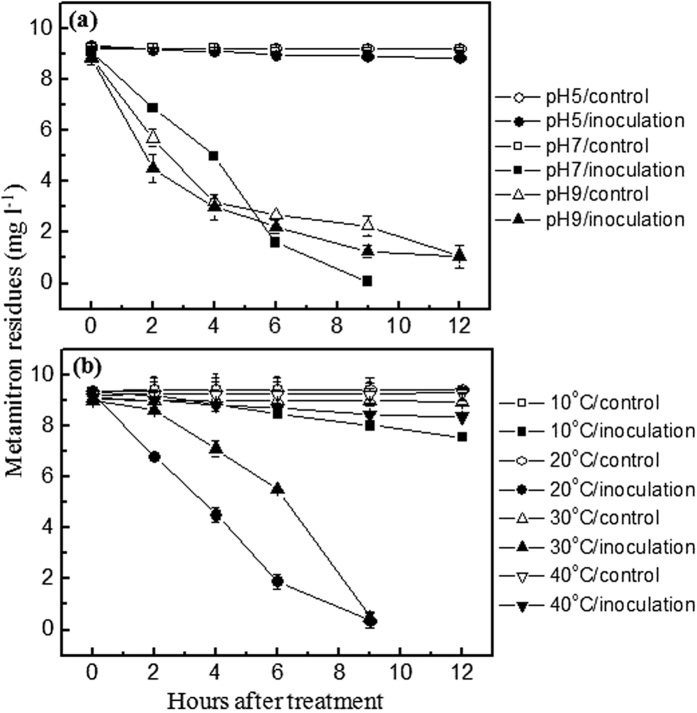
Effect of pH (**a**) and temperature (**b**) on the degradation of metamitron at the concentration of 10 mg/l by the strain MET in mineral salts medium.

Figure 3a and Table S7 show the effect of pH on the degradation of 10 mg/l of metamitron by the isolate MET in MSM at 20 °C and 150 rpm. After 9 h of incubation, the dissipation rates of metamitron in the control without MET inoculation at pH 5.0, 7.0, and 9.0 were 0.01%, 0.5%, and 74.7%, respectively, indicating that metamitron was stable in both acidic and neutral solutions but not in the alkaline solution (Fig. 3a). Approximately 4.2%, 99.2%, and 85.6% of metamitron was degraded by the isolate MET at pH 5.0, 7.0, and 9.0, respectively, after 9 h of incubation in the dark (Fig. 3a). As shown in Table 2, the degradation rates of metamitron by the isolate MET at pH 5.0, 7.0, and 9.0 were 0.04, 1.00, and 0.10 mg/l/h, with the corresponding degradation half-lives of 144.4, 1.5, and 52.5 h, respectively, after 9 h of incubation. The results showed that the degradation of metamitron by the isolate MET was significantly (*p* ≤ 0.05) faster at pH 7.0 than at pH 5.0 and 9.0, implying that microbial degradation was a pH-dependent process^20^,^21^,^22^. Similarly, Liu *et al*.^23^ reported that the optimal pH for the degradation of the herbicide butachlor by *Rhodococcus* sp. B1 was 7.0–7.5. Kolekar *et al*.^24^ found that the maximum removal of the triazine herbicide atrazine by *Rhodococcus* sp. BCH2 occurred in liquid medium at pH 7.0. Zhang *et al*.^10^ reported the maximum degradation of metribuzin by *Bacillus* sp. N1 at pH 7.0.

### Effect of temperature on metamitron biodegradation

The effect of temperature on the degradation of 10 mg/l of metamitron by the isolate MET in MSM at pH 7.0 is shown in Fig. 3b and Table S8. The dissipation rate of metamitron was less than 1.69% in all of the controls without the isolate MET inoculation. As shown in Table 2, the degradation rates of metamitron by the isolate MET at 10, 20, 30, and 40 °C were 0.15, 0.77, 0.75, and 0.06 mg/l/h, with the corresponding degradation half-lives of 36.3, 4.2, 7.8, and 95.0 h, respectively. The results showed that the optimal temperature range for metamitron biodegradation was 20–30 °C. Some researchers also found that temperature had a significant influence on the biodegradation of triazinone herbicides and other herbicides^9^,^25^,^26^. Liu *et al*.^23^ reported that the optimal temperature for the degradation of chloroacetamide herbicides by *Rhodococcus* sp. B1 was 30 °C. The fast degradation of atrazine by *Rhodococcus* sp. BCH2 was observed at 30 °C^24^. Zhang *et al*.^10^ observed the maximum degradation of metribuzin by *Bacillus* sp. N1 at 30 °C.

### Biodegradation pathway of metamitron revealed by HPLC-MS/MS analysis

**Figure 4:**
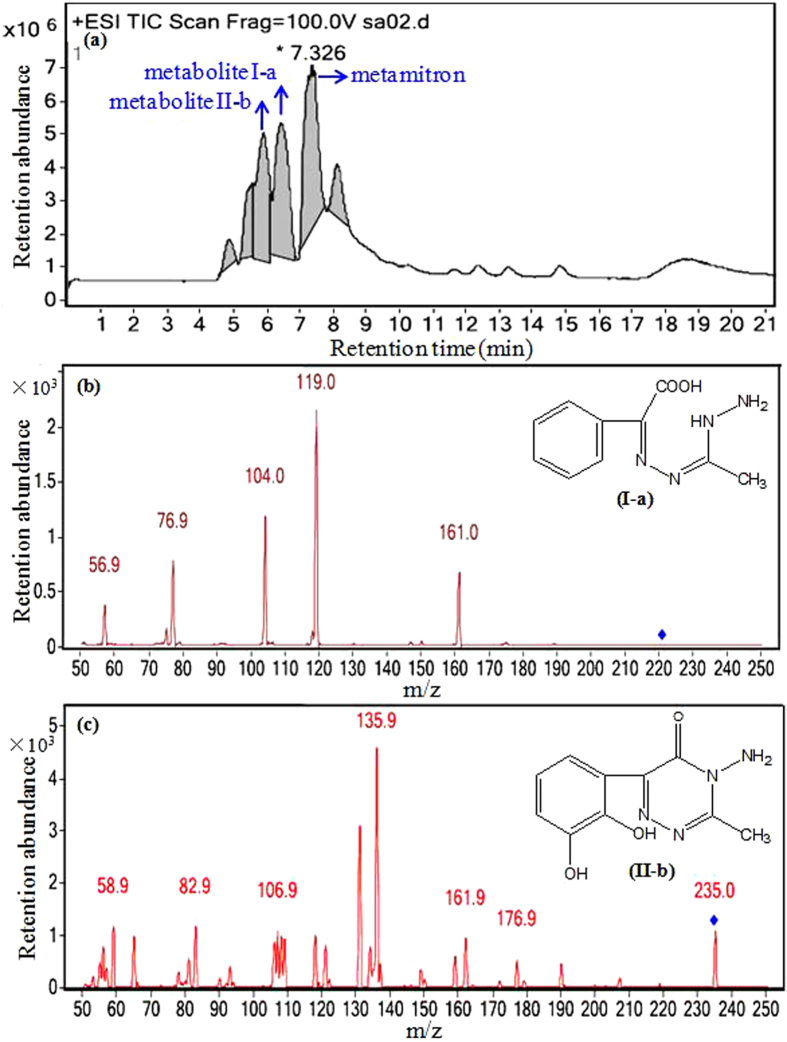
Total ion chromatogram of metamitron degradation by the isolate MET (**a**), mass spectra and the proposed structures of metamitron metabolites I-a (**b**) and II-b (**c**) in distilled water using extraction method I.

**Figure 5:**
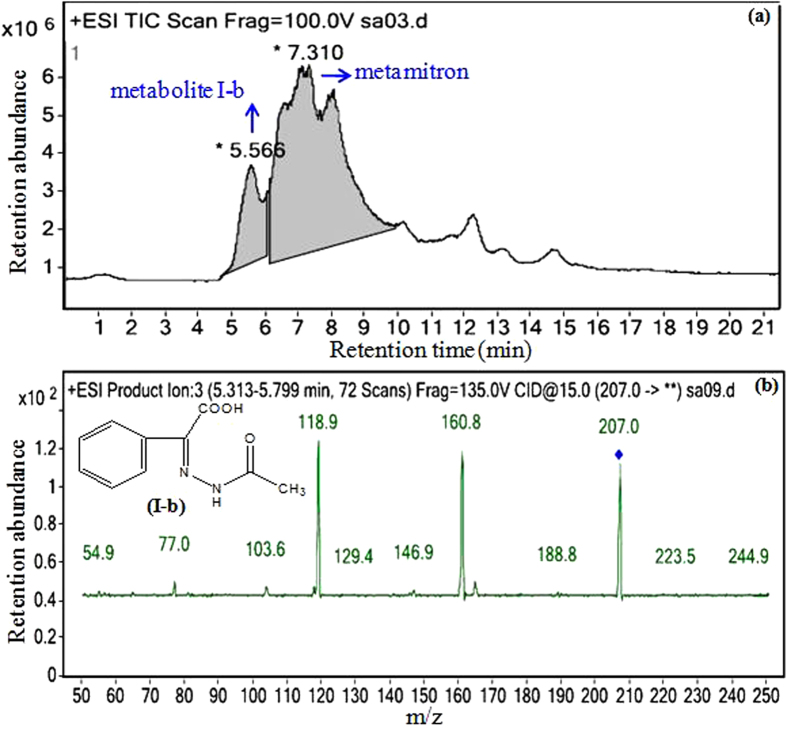
Total ion chromatogram of metamitron degradation by the isolate MET (**a**), mass spectrum and the proposed structure of metamitron metabolite I-b (**b**) in mineral salts medium using extraction method II.

The total ion chromatograms and mass spectra of the metamitron metabolites produced by the isolate MET in distilled water and in MSM are shown in Figs 4 and 5, respectively. After 48 h of inoculation with the isolate MET, two metamitron metabolites were identified with mass ions at *m/z* 221 and 235 in distilled water (Fig. 4a) and one metamitron metabolite was identified with a mass ion at *m/z* 207 in MSM by HPLC-MS/MS (Fig. 5a). Based on the known standard compounds and reported metamitron metabolites, these three metabolites were proposed to be 2-(3-hydrazinyl-2-ethyl)-hydrazono-2-phenylacetic acid (HPA, C_10_H_12_N_4_O_2_, Fig. 4b), 2,3-dihydroxymetamitron (DHM, C_10_H_10_N_4_O_3_, Fig. 4c) and methyl benzoylformate acetylhydrazone (MBA, C_10_H_10_N_2_O_3_, Fig. 5b), respectively. The compounds corresponding to the other peaks in the total ion chromatograms were not identified and might not be metamitron metabolites produced by the isolate MET. Additionally, the accumulation of these metabolites was not observed by the determination in distilled water or in MSM during incubation for 48 h, suggesting that the isolate MET could mineralize metamitron with the identified metabolites acting as intermediates.

**Figure 6:**
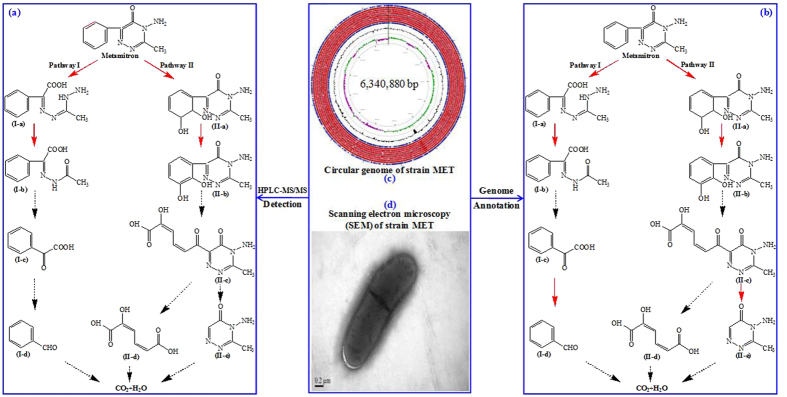
The potential degradation pathway of metamitron by the isolate MET based on genome annotation (**a**), the proposed degradation pathway of metamitron by the isolate MET based on HPLC-MS/MS analysis (**b**), circular genome of the isolate MET (**c**), and scanning electron microscopy (SEM) of the isolate MET (**d**). The reaction indicated by the full line and the dotted line represented the detected degradation step and the undetected degradation step, respectively. I-a: 2-(3-hydrazinyl-2-ethyl)-hydrazono-2-phenylacetic acid (HPA); I-b: methyl benzoylformate acetylhydrazone (MBA); I-c: benzoylformic acid (BA); I-d: benzaldehyde; II-a: 2,3-dihydro-2,3-dihydroxymetamitron (DDHM); II-b: 2,3-dihydroxymetamitron (DHM); II-c: 3-methyl-4-amino-6(2-hydroxy-muconic acid)-1,2,4-triazine-5(4H)-one (MAHT); II-d: 2-hydroxy-muconic acid; and II-e: 3-methyl-4-amino-1,2,4-triazine-5(4H)-one (MAT).

According to the structures of the metabolites identified in this study, the following two mineralization pathways via cleavage of the triazinone ring or phenyl ring could be proposed: 1) metamitron is initially converted to HPA by hydrolytic cleavage of the amide bond in the triazinone ring, and subsequently to MBA by deamination, transformed to BA and benzaldehyde, and ultimately mineralized to carbon dioxide (Pathway I in Fig. 6a) and 2) metamitron is initially converted to 2,3-dihydro-2,3-dihydroxymetamitron (DDHM) by hydroxylation, and subsequently to DHM by dehydrogenation, which was further cleaved by *meta*-fission in the phenyl ring and hydrolysis, and finally mineralized to carbon dioxide (Pathway II in Fig. 6a).

Parekh *et al*.^12^ isolated the bacterial strain *Rhodococcus* sp. 0246b, which was capable of degrading metamitron via partial mineralization of the phenyl ring. However, cleavage of the triazinone ring was not found in *Rhodococcus* sp. 0246b-mediated metamitron degradation. Accordingly, this study is the first report that a bacterial strain could simultaneously mineralize metamitron by two degradation pathways via cleavage of both the triazinone ring and phenyl ring. Some bacterial strains from genus *Rhodococcus* (the same genus in which we placed the isolate MET) could degrade other herbicides by other reaction modes^27^,^28^. *Rhodococcus* sp. FJ1117YT could transform methylthio-*s*-triazines into 2-hydroxy derivatives via sulfur oxidation^11^. *Rhodococcus* sp. B1 could degrade chloroacetamide herbicides by N-dealkylation^23^. Additionally, deamino-metamitron and metamitron-N-glucoside were found as primary products of metamitron metabolism in Chenopodium album^3^.

### Biodegradation pathway of metamitron revealed by genome annotation

Potential degradation genes that hit the isolate MET genome are summarized in Table S4. As shown in Table S4, the identified MDGs-I in the isolate MET genome played roles in the conversion of metamitron to HPA by hydrolysis (*hdl* gene encoding a hydrolase), HPA to MBA by deamination (*dan* gene encoding a deaminase), and BA to benzaldehyde by decarboxylation (*dcl* gene encoding a decarboxylase). The identified MDGs-II in the isolate MET genome was responsible for the conversion of metamitron to DDHM by hydroxylation (*hdx* gene encoding a hydroxylase), DDHM to DHM by dehydrogenation (*dhn* gene encoding a dehydrogenase), 3-methyl-4-amino-6(2-hydroxy-muconic acid)-1,2,4-triazine-5(4H)-one (MAHT) to 3-methyl-4-amino-1,2,4-triazine-5(4H)-one (MAT) by hydroxylation (*hdl* gene encoding a hydrolase). As shown in Fig. 6b, the two potential metamitron degradation pathways by the isolate MET were revealed by a BLAST search against the MDGs-I and MDGs-II databases. Pathway I showed that the isolate MET converted metamitron into HPA by hydrolysis of the triazinone ring, and then transformed to MBA, BA and benzaldehyde by deamination, and ultimately mineralized to carbon dioxide; Pathway II showed that the isolate MET transformed metamitron into DDHM and DHM by hydroxylation and dehydrogenation of the phenyl ring, and then to MAHT and MAT by meta-fission in the phenyl ring and hydroxylation, and finally mineralized to carbon dioxide. Similar to the genome analysis method, the microbial degradation mechanism of another triazine herbicide atrazine was also revealed using metagenomic analysis by Fang *et al*.^29^, who found the potential nearly complete biodegradation pathway of atrazine in sediments by dechlorination, dealkylation, deamination, hydroxylation, and s-triazine ring cleavage reactions. In this study, the functions of the identified degradation genes were not verified by other methods. Therefore, further studies need to be conducted to confirm the functions of these identified potential degradation genes.

## Methods

### Chemicals

A standard sample of metamitron (purity 98.0%) was purchased from Dr. Ehrenstorfer GmbH (Augsburg, Germany). Analytical grade dichloromethane, sodium chloride and anhydrous sodium sulfate were purchased from Sinopharm Chemical Reagent Co. Shanghai, China. Chromatographic grade methanol was purchased from Tedia Co., USA.

### Isolation and identification of microorganism

Activated sludge samples were collected from a pesticide wastewater treatment plant located in Hefei, Anhui, China. Samples were homogenized and then divided into three subsamples. Each subsample was mixed thoroughly with 100 ml of distilled water in a 250-ml Erlenmeyer flask. Subsequently, a 1.0-ml aliquot from the suspension was inoculated into a 100-ml Erlenmeyer flask containing 20 ml of sterile mineral salts medium (MSM) (MgSO_4_·7H_2_O, 0.40 g; FeSO_4_·7H_2_O, 0.002 g; K_2_HPO_4_, 0.20 g; (NH_4_)_2_SO_4_, 0.20 g; CaSO_4_, 0.08 g; and H_2_O, 1000 ml; pH 7.0) supplemented with 1.0 mg/l of metamitron as the sole carbon and energy source. Each culture was incubated for 1 week on a rotary shaker at 30 °C and 150 rpm. Subsequently, the culture was repeatedly acclimated in fresh sterile MSM with increasing concentrations of metamitron from 5.0 to 20.0 mg/l. Eventually, one pure bacterium MET was isolated and then identified by morphological characteristics and 16S rDNA sequencing. Carbon substrate utilization by the isolate MET was assessed using the BIOLOG system (BIOLOG Inc., Hayward, CA, USA) according to the method described in Supplementary Information.

### Effect of concentration, temperature, and pH on metamitron biodegradation in pure culture

To evaluate the ability of the strain MET to degrade metamitron, the experiment was conducted in a 100-ml Erlenmeyer flask containing 20 ml of sterilized MSM supplemented with metamitron at a certain concentration as the sole carbon and energy source. Each flask was inoculated with a suspension of the strain MET to give a biomass level of OD_610_ = 0.1. All flasks were incubated in a rotary shaker at 30 °C and 150 rpm in the dark. At 0, 2, 4, 6, and 12 h after inoculation, the whole culture was sampled to determine for the metamitron concentration. The treatment without inoculation of the strain MET under the same conditions was used as the control. Each treatment was performed in triplicate.

To examine the effect of the initial metamitron concentration on its biodegradation, the MSM was fortified with three metamitron concentrations (1, 10 and 100 mg/l). To estimate the effect of pH on metamitron biodegradation, the MSM was prepared with NaH_2_PO_4_-Na_2_HPO_4_ buffer (0.1 mol/l) at pH 5.0, 7.0, and 9.0, respectively. To investigate the effect of temperature on metamitron biodegradation, the cultures were incubated at 10, 20, 30, and 40 °C, respectively.

### Extraction and determination of metamitron and its metabolites

The metamitron degradation solution for the strain MET in 20 ml of MSM medium was transferred to a 250-ml separatory funnel and extracted 3 times with 50 ml of dichloromethane. The organic phase was passed through anhydrous sodium sulfate and collected in a 250-ml flat bottom flask. The extract was concentrated to approximately 1 ml on a rotary evaporator, dried under a gentle nitrogen stream, and then dissolved in 10 ml of methanol for the determination by HPLC.

To obtain sufficient quantities of the possible metamitron metabolites produced by the strain MET, the experiment was conducted in a 100-ml Erlenmeyer flask containing 20 ml of MSM supplemented with 200 mg/l of metamitron. Each flask was inoculated with 0.5 ml of the strain MET suspension to give a biomass level of OD_610_ = 0.1. All flasks were incubated in a rotary shaker at 30 °C and 150 rpm in the dark. At 0, 6, 12, 24, 36, and 48 h after inoculation, two methods were used to extract the metamitron metabolites: 1) the whole culture was passed through a 0.2 μm microporous membrane and then the metamitron metabolites were determined and 2) the whole culture was decanted into a 250-ml separatory funnel and extracted 3 times with 50 ml of dichloromethane. Then, the organic phase was passed through anhydrous sodium sulfate and collected in a 250-ml flat bottom flask. The extract was concentrated to approximately 1 ml on a rotary evaporator under vacuum condition, dried under a gentle nitrogen stream, and finally dissolved in 10 ml of methanol for the determination of the metamitron metabolites. The metamitron analysis was performed using an Agilent 1200 HPLC (Agilent Technologies, USA) equipped with a diode array detector (DAD) and an Eclipse XDB-C_18_ stainless steel column (150 mm × 4.6 mm i.d., 5 μm, Agilent Technologies, USA) according to the method described in Supplementary Information.

### Library construction and genome sequencing

Genomic DNA was extracted from 1 ml of the isolate MET suspension using a QIAamp DNA mini kit (Qiagen, Hilden, Germany) according to the manufacturer’s protocol. The concentration and quality of the extracted DNA were determined using a NanoDrop 2000 spectrophotometer (Thermo Scientific, Wilmington, DE, USA) and gel electrophoresis. The DNA was mechanically fragmented by ultrasonic lysis and then gel size-selected for ~500 base pairs (bp) fragment size. Illumina fragment libraries were constructed using Illumina paired-end DNA sample prep kit v1 with some modifications^30^. The overhangs from the fragmentation were converted into blunt ends using the T4 DNA polymerase, Klenow Fragment, and T4 Polynucleotide Kinase (New England Biolabs, MA, USA). Sequencing adapters were ligated to the ends of the end-repaired DNA fragments. All reaction clean-ups were performed using a MinElute PCR purification kit (Qiagen, Hilden, Germany). Size-selected DNA was purified with a Qiagen MinElute gel extraction kit and quantified using the Quant-it dsDNA HS assay kit (Invitrogen). Subsequently, the constructed DNA libraries was used for paired-end sequencing using an Illumina Hiseq 2500 sequencing platform (Biomarker, Beijing, China)^31^.

### Database construction

Metamitron degradation primarily included the triazine ring cleavage pathway (Pathway I) and the phenyl ring cleavage pathway (Pathway II). Protein databases of metamitron degradation genes containing 5 sub-databases (*hdl, dan, cbe, dcl*, and *prc*) for Pathway I and 5 sub-databases (*hdx, dhn, mfe, hdl*, and *trc*) for Pathway II were retrieved from the NCBI GenBank database. The metamitron Pathway I degradation gene (MDGs-I) and Pathway II degradation gene (MDGs-II) databases were deduplicated to obtain 2210 and 3809 non-redundant proteins, respectively (Table S9).

### Bioinformatic analysis

For quality control of the sequencing data, the raw reads were filtered using a self-written script to remove low quality reads (three or more ambiguous nucleotides and a quality value < 20). Adapter sequences with at least a 15 bp overlap between the adapter and reads that allowed 3 bp mismatches were discarded. All reads were further cleaned to remove duplicated sequences using a self-written script. Then, the paired-end high quality reads were *de novo* assembled into contigs with SOAPdenovo2^32^ using a Kmer length of 71 bp^33^, which showed good assembly statistics compared to the other lengths. The single base error of the assembly result was corrected using mapping information. Contigs over 500 bp in length were considered for the downstream analysis. The obtained contigs were aligned against the established metamitron degradation genes databases using BLASTx with an E-value cut-off of 10^−6^, and then the best hit results were filtered with the cutoff at an identity ≥80% and alignment length ≥25 aa using a self-written Python script.

The draft genome was annotated by the GeneMarkS + tool according to the NCBI Prokaryotic Genome Annotation Pipeline (PGAP)^34^. The genomic functional annotation of the isolate MET was conducted by a Reversed Position Specific BLAST (RPS BLAST) program search against the Clusters of Orthologous Groups (COG), Pfam, Swiss-Prot, TrEMBL, and nr databases, respectively, with an E-value cutoff of 1e^−10^. Kyoto Encyclopedia of Genes and Genomes (KEGG) mapping of the isolate MET was conducted against the KEGG pathway database^35^, and the cellular components, molecular functions, and biological processes of the isolate MET were annotated using an InterProScan search against the Gene Ontology (GO) database. All obtained contigs were joined together by the CLC Main Workbench (version 7.6.2, CLC Bio, Aarhus, Denmark) and then submitted to the CGView Server to plot a graphical circular map of the isolate MET (http://stothard.afns.ualberta.ca/cgview_server/). Orthologous protein sequences of the isolate MET were classified by family, and unique gene families were found using OrthoMCL software (version 2.0)^36^. Gene sequences of the isolate MET and relatively close species were compared to find homologous genes using BLASTn, and then the Ka/Ks values of the homologous genes were calculated using the KaKs_Calculator software (version 2.0) to determine the size of the selection pressure^37^. Collinearity analysis was performed based on the protein sequences of the isolate MET and the near edge species.

### Nucleotide sequence deposite

The isolate MET was submitted to the China Center for Type Culture Collection (CCTCC) under M 2014116. The isolate MET 16S rDNA sequence was deposited in the NCBI GenBank database under accession number KX545422. The whole genome shotgun project was deposited at DDBJ/EMBL/GenBank under accession number LNIU00000000 (submission ID SUB1181896, BioProject ID PRJNA301801, and BioSample ID SAMN04260011).

## Additional Information

**How to cite this article**: Fang, H. *et al*. Characterization and genome functional analysis of a novel metamitron-degrading strain *Rhodococcus* sp. MET via both triazinone and phenyl rings cleavage. *Sci. Rep.* **6**, 32339; doi: 10.1038/srep32339 (2016).

## Supplementary Material

Supplementary Information

## Figures and Tables

**Table 1:** Genome properties of the isolate MET.

Property	Value	Property	Value
Raw reads size (bp)	2,050,913,844	CDS	5762
Clean reads size (bp)	1,743,276,767	Total size (bp)	5,816,232
Genome size (bp)	6,340,880	Mean length (bp)	971
Fold coverage	275×	miRNA numbers	11
Scaffold number	30	miRNA family	11
Scaffold N50 (bp)	632,855	rRNA numbers	196
Contig number (> 500 bp)	69	rRNA family	3
Contig length (bp)	6,336,286	tRNA numbers	35
Contig N50 (bp)	184,392	tRNA family	31
GC content (%)	62.47	Protein-coding genes	5987
COG annotation	4,075	Pseudogene	6
GO annotation	4,072	Kmer depth	71
KEGG annotation	2,423	ncRNAs	1
Pfam annotation	2,722	5S rRNAs	1
Swiss-Prot annotation	3,794	16S rRNAs	1
TrEMBL annotation	4,376	23S rRNAs	1
nr annotation	5,906	TRF repetitive sequence number	370
All annotated genes	5,907	TRF repetitive sequence length (bp)	16,758
Total gene number in gene family	5,540	Gene in unique gene family	23
Family number	5,371	Gene not in gene family	447
Unique gene family	9	Total unique gene	470

**Table 2:** Kinetic data of metamitron degradation by the strain MET.

Concentration (mg/l)	pH	Temperature	Kinetic equation	Degradation rate (mg/l/h)	DT_50_^a^ (h)	*R*^2^
1	7	20	*C* = 0.8607 e^−1.0396*t^	0.14	0.7 i^b^	0.97
10	7	20	*C* = 10.1231 e^−0.3954*t^	1.22	1.8 h	0.89
100	7	20	*C* = 97.2159 e^−0.0099*t^	0.97	70.0 c	0.99
10	5	20	*C* = 9.28726 e^−0.0048*t^	0.04	144.4 a	0.97
10	7	20	*C* = 16.6020 e^−0.4705*t^	1.00	1.5 h	0.88
10	9	20	*C* = 8.9691 e^−0.0132*t^	0.10	52.5 d	0.92
10	7	10	*C* = 9.5171 e^−0.0191*t^	0.15	36.3 e	0.98
10	7	20	*C* = 9.4132 e^−0.1666*t^	0.77	4.2 g	0.96
10	7	30	*C* = 9.2478 e^−0.0889*t^	0.75	7.8 f	0.84
10	7	40	*C* = 9.1155 e^−0.0073*t^	0.06	95.0 b	0.99

^a^Degradation half-lives.

^b^Data followed by a different letter in the same column are significantly different (*p* ≤ 0.05).
