# Supplementary material for: Characterization and genome functional analysis of a novel metamitron-degrading strain Rhodococcus sp. MET via both triazinone and phenyl rings cleavage
Source: Sci Rep. 2016 Aug 31;6:32339. doi: 10.1038/srep32339 (PMC5006018; doi:10.1038/srep32339)
Supplement: Supplementary Information [file srep32339-s1.doc]

**Supplementary Information**

**Characterization and genome functional analysis of a novel metamitron-degrading strain *Rhodococcus* sp. MET via both triazinone and phenyl rings cleavage**

Hua Fang, Tianheng Xu, Duantao Cao, Longyin Cheng, Yunlong Yu*

Institute of Pesticide and Environmental Toxicology, College of Agricultural and Biotechnology, Zhejiang University, Hangzhou 310058, China. Correspondence and requests for materials should be addressed to Y.L.Y. (email: ylyu@zju.edu.cn)

**List of Contents:**

**SI Section I**

BIOLOG assay

DNA extraction, 16S rDNA amplification and sequencing

Determination of metamitron and its metabolites

**SI Section II**

**Table S1** Metabolic profile of the isolate MET

**Table S2** 16S rDNA sequence of the isolate MET

**Table S3** Genome functional annotation of the isolate MET against GO database

**Table S4** Potential degradation genes that hit the isolate MET genome

**Table S5** Rapid evolutionary gene detection

**Table S6** Degradation of metamitron at concentrations of 1, 10, and 100 mg/l by the isolate MET in mineral salts medium at pH 7.0 and 30 ºC

**Table S7** Effect of pH on the degradation of metamitron at the concentration of 10 mg/l by the isolate MET in mineral salts medium

**Table S8** Effect of temperature on the degradation of metamitron at the concentration of 10 mg/l by the isolate MET in mineral salts medium

**Table S9** Composition of metamitron degradation genes database

**Fig. S1** Collinearity analysis on the protein sequences between the isolate MET and the closely related species *Rhodococcus opacus* strain B4

**Fig. S2** Venn diagram on genomic comparison of the isolate MET and its close strains RHA1, PR4, B4 and ATCC 4277

**SI Section I**

**BIOLOG assay.** Carbon substrate utilization by the isolate MET was assessed using the BIOLOG system (BIOLOG Inc., Hayward, CA, USA) according to the method described in Supplementary Information. The isolate MET was incubated on BUG (BIOLOG Universal Growth Agar) at 30 °C for 24 h prior to the assay. Fresh colonies were removed from the BUG medium with a long cotton swab and suspended in 15 ml of inoculation fluid with turbidity equivalent to 72% transmittance as measured by the BIOLOG turbidimeter. Each well in the BIOLOG GP2 microplate^TM^ was inoculated with 150 μl of the bacterial suspension, and then incubated in a biochemical incubator at 25 ± 1 °C. Color development in the wells was monitored at 6 h and 24 h using a microplate reader with a 590 nm filter (BIO-TEK Instruments, Winooski, VT, USA). The output data were recorded by the automatic threshold option using the BIOLOG software (BIOLOG Gen III database v2.7).

**DNA extraction, 16S rDNA amplification and sequencing.** The genomic DNA was extracted from 1 ml of suspension of the isolate MET using a QIAamp DNA mini kit (Qiagen, Hilden, Germany) according to the manufacturer's protocol. The concentration and quality of the extracted DNA were determined using a NanoDrop 2000 spectrophotometry (Thermo Scientific, Wilmington, DE, USA) and gel electrophoresis. The 16S rDNA was amplified using the polymerase chain reaction (PCR) with genomic DNA as template and a pair of universal primers, 27F (5'-AGAGTTTGATCCTGGCTCAG-3') and 1492R (5'-GGTTACCTTGTTACGACTT-3'). The PCR reaction system contained 25.0 μl, which included 1.0 μl of 20 ng/μl template, 2.5 μl of buffer (10×), 2.0 μl of 2.5 mM dNTP, 1.0 μl of each primer (10 μM), 0.25 μl of Taq polymerase, and 18.25 μl of ultra-pure water. The PCR conditions were as follows: initial denaturation at 94 °C for 5 min, followed by 31 cycles of denaturation at 94 °C for 30 s, annealing at 58 °C for 45 s, and extension at 72 °C for 45 s, and a final extension at 72 °C for 5 min. The PCR products were purified using QIAGEN PCR Purification kit (Qiagen) and sequenced by the dideoxy chain termination method on a model 373 automated DNA sequencer (Applied Biosystems). Sequence data were compared with other bacterial 16S rDNA gene sequences in the GenBank database by Blast searching.

**Determination of metamitron and its metabolites.** The metamitron analysis was performed using an Agilent 1200 HPLC (Agilent Technologies, USA) equipped with a diode array detector (DAD) and an Eclipse XDB-C_18_ stainless steel column (150 mm×4.6 mm i.d., 5 μm, Agilent Technologies, USA). A 10 μl aliquot of each sample was detected by measuring the absorbance at 281 nm with an elution of the mixture of methanol and water (45:55, v/v) at a flow rate of 0.8 ml/min.

The metamitron metabolites were identified using an Agilent 1290 HPLC coupled with an Agilent 6460 tandem triple quadrupole mass spectrometer equipped with an ESI source operating in the positive ionization (PI) mode (Agilent Technologies, USA). An Eclipse XDB-C_18_ stainless steel column (150 mm×4.6 mm i.d., 5 μm, Agilent Technologies, USA) was used for the separation of the metamitron metabolites. A mixture of methanol and water (45:55, v/v) was used as the mobile phase at a flow rate of 0.3 ml/min. For the fragmentation experiments with the ESI source, sample solutions eluted from the HPLC column were introduced into the ion source via a metal ESI needle set at high voltage (3.0 KV) and a heated capillary at 350 °C. The other instrument conditions were as follows: the nebulizer pressure was 45 psi, the drying gas flow rate was 11 l/min, and the drying gas temperature was 325 °C. The full scan mode was utilized in the mass range of 50 to 320 *m/z*.

**SI Section II**

**Table S1** Metabolic profile of the isolate MET

| Substrate (reaction) | Substrate (reaction) | Substrate (reaction) |
| --- | --- | --- |
| Water (-) | α-cyclodextrin (-) | β-cyclodextrin (-) |
| N-acetyl-D-glucose (-) | Starch (-) | Synanthrin (-) |
| D-galacturonic acid (-) | Tween 40 (+) | Tween 80 (+) |
| N-acetyl-galactose (+) | Dextrin (+) | Amygdalin (-) |
| 3-methyl-D-galactose (-) | D-Arabinose (+) | Arbutin (-) |
| α-methy-D-glucoside (-) | D-fructose (+) | L-trehalose (+) |
| α-methyl-D-galactose (-) | Mannan (-) | Gentiobiose (+) |
| D-gluconic acid (+) | α-D-glucose (+) | m-inositol (+) |
| α-methyl-D-mannose (-) | Lactulose (-) | Maltose (+) |
| γ –hydroxybutyric acid (+) | D-mannitol (-) | D-mannitol (+) |
| Acyl-L-amide glutamine (-) | D-melibiose (-) | D-galactose (+) |
| β-methyl-D-galactose (-) | L-Arabinose (+) | D-cellobiose (+) |
| β-methyl-D-glucose (-) | α-D-lactose (-) | D-ribose (+) |
| D-allulose psicose (+) | D-melitriose (-) | L-rhamnose (+) |
| 1- phosphoric -α-D-glucose (-) | Saligenin (-) | Sedoheptulosan (-) |
| 6-phosphoric-D-levulose (-) | Lupeose (-) | Sucrose (+) |
| 6- phosphoric -D-glucose (-) | D-trehalose (-) | Turanose (+) |
| 5’-single uridine phosphate (-) | D-xylose (+) | Acetic acid (+) |
| α-hydroxybutyric acid (+) | Lactamide (-) | Maltotriose (+) |
| p-hydroxyphenylacetic acid (-) | α-oxoglutarate (+) | α-ketoglutaric acid (+) |
| β- hydroxybutyric acid (+) | D-methyl lactate (+) | L-lactic acid (+) |
| L-pyroglutamic acid (-) | L-malic acid (+) | Methyl pyruvate (+) |
| Succnic acid dimethyl ester (+) | Propionic acid (+) | Pyruvic acid (+) |
| Succinamic acid (+) | Succinic acid (+) | D-melezitose (-) |
| L-alanine amine (+) | D-alanine (+) | L-alanine (+) |
| L-proplence ammonia glycine (+) | L-asparagine (+) | L-glutamic acid (+) |
| glycyl-L- glutamic acid (+) | D-malic acid (+) | L-serine (+) |
| D-L-α-phosphoglycerol (-) | 2,3-butanediol (-) | Glycerol (+) |
| 2'-deamination adenosine (-) | Adenosine (+) | Inosine (+) |
| 5'-single adenosinephospate (-) | Uridine (-) | Thymidine (-) |
| 5’-single thymidylic phosphate (-) | Xylitol (-) | D-sorbitol (-) |
| 6-O-D-pyran glucose acyl furan-D-fructose (+) | D-tagatose (+) | Butanediamine (-) |

+: growing well；-: no growth.

**Table S2** 16S rDNA sequence of the isolate MET

| 16S rDNA size (bp) | 1127 |
| --- | --- |
| Similarity | 99% |
| Accession number | KX545422 |
| Putative genus | *Rhodococcus* |
| **16S rDNA sequence:**  AAGGTCAGGATCAACTCTACTGCTCCTCCCACAACCGGGTTAAGCCACCGGCTTCGGGTGTTACCGACTTTCATGACGTGACGGGCGGTGTGTACAAGGCCCGGGAACGTATTCACCGCAGCGTTGCTGATCTGCGATTACTAGCGACTCCGACTTCACGGGGTCGAGTTGCAGACCCCGATCCGAACTGAGACCAGCTTTAAGGGATTCGCTCCACCTCACGGTCTCGCAGCCCTCTGTACTGGCCATTGTAGCATGTGTGAAGCCCTGGACATAAGGGGCATGATGACTTGACGTCGTCCCCACCTTCCTCCGAGTTGACCCCGGCAGTCTCTTACGAGTCCCCACCATAACGTGCTGGCAACATAAGATAGGGGTTGCGCTCGTTGCGGGACTTAACCCAACATCTCACGACACGAGCTGACGACAGCCATGCACCACCTGTATACCGACCACAAGGGGGGCCACATCTCTGCAGCTTTCCGGTATATGTCAAACCCAGGTAAGGTTCTTCGCGTTGCATCGAATTAATCCACATGCTCCGCCGCTAGTGCGGGCCCCCGTCAATTCCTTTGAGTTTTAGCCTTGCGGCCGTACTCCCCAGGCGGGGCGCTTAATGCGTTAGCTACGGCACGGATTCCGTGGAAGGAACCCACACCTAGCGCCCACCGTTTACGGCGTGGACTACCAGGGTATCTAATCCTGTTCGCTACCCACGCTTTCGTTCCTCAGCGTCAGTTACTGCCCAGAGACCCGCCTTCGCCACCGGTGTTCCTCCTGATATCTGCGCATTTCACCGCTACACCAGGAATTCCAGTCTCCCCTGCAGTACTCAAGTCTGCCCGTATCGCCTGCAAGCCAGCAGTTGAGCTGCTGGTTTTCACAAACGACGCGACAAACCGCCTACGAACTCTTTACGCCCAGTAATTCCGGGACAACGCTTGCACCCTACGTATTACCGCGGCTGCTGGCACGTAGTTAGCCGGTGCTTCTTCTGCAGTACCGTCACTTGCGCTTCGTCCCTGCTGAAAGAGGTTTACACCCGAAGCCGTCATCCCCTCACGCGCGTCGCTGCATCAGCTTCGCCCATTGTGCATATTCCCCACTGCTGCCTCCCGTAGGAGTCT | |

**Table S3** Genome functional annotation of the isolate MET against GO database

| **GO classify** | **Gene function** | **Gene number** |
| --- | --- | --- |
| cellular component | extracellular region | 11 |
| cellular component | nucleoid | 3 |
| cellular component | membrane | 318 |
| cellular component | macromolecular complex | 140 |
| cellular component | organelle | 54 |
| cellular component | extracellular region part | 1 |
| cellular component | organelle part | 16 |
| cellular component | membrane part | 492 |
| cellular component | cell part | 670 |
| **Total** | | **1705** |
| molecular function | protein binding transcription factor activity | 20 |
| molecular function | nucleic acid binding transcription factor activity | 235 |
| molecular function | catalytic activity | 2831 |
| molecular function | receptor activity | 39 |
| molecular function | structural molecule activity | 61 |
| molecular function | transporter activity | 376 |
| molecular function | binding | 1890 |
| molecular function | electron carrier activity | 60 |
| molecular function | antioxidant activity | 40 |
| molecular function | metallochaperone activity | 2 |
| molecular function | enzyme regulator activity | 9 |
| molecular function | protein tag | 1 |
| molecular function | nutrient reservoir activity | 1 |
| molecular function | molecular transducer activity | 100 |
| **Total** | | **5665** |
| biological process | Reproduction | 1 |
| biological process | cell killing | 2 |
| biological process | metabolic process | 2452 |
| biological process | cellular process | 1581 |
| biological process | biological adhesion | 5 |
| biological process | developmental process | 1 |
| biological process | growth | 1 |
| biological process | single-organism process | 1889 |
| biological process | response to stimulus | 251 |
| biological process | localization | 496 |
| biological process | multi-organism process | 6 |
| biological process | biological regulation | 634 |
| biological process | cellular component organization or biogenesis | 34 |
| **Total** | | **7353** |

**Table S4** Potential degradation genes that hit the isolate MET genome

| **Scaffold no.** | **Subject id** | **Alignment length** | **Enzyme** | **Degradation step** |
| --- | --- | --- | --- | --- |
| **Metamitron degradation genes for pathway I (MDGs-I)** | | | | |
| Scaffold 1 | KIL05529.1 | 255 | decarboxylase | BA-benzaldehyde |
| Scaffold 2 | ERI43401.1 | 691 | hydrolase | Metamitron-HPA |
| Scaffold 5 | ERI40911.1 | 255 | hydrolase | Metamitron-HPA |
| Scaffold 11 | ERI41656.1 | 303 | hydrolase | Metamitron-HPA |
| Scaffold 13 | EQB15891.1 | 517 | decarboxylase | BA-benzaldehyde |
| Scaffold 18 | EQB18413.1 | 316 | hydrolase | Metamitron-HPA |
| Scaffold 19 | ERI44187.1 | 700 | hydrolase | Metamitron-HPA |
| Scaffold 23 | ERI40669.1 | 459 | decarboxylase | BA-benzaldehyde |
| Scaffold 24 | EQB15060.1 | 205 | deaminase | HPA-MBA |
| Scaffold 26 | KIL03820.1 | 120 | hydrolase | Metamitron-HPA |
| Scaffold 27 | ERI41558.1 | 630 | deaminase | HPA-MBA |
| Scaffold 28 | ERI42602.1 | 1133 | decarboxylase | BA-benzaldehyde |
| Scaffold 30 | ERI41906.1 | 733 | hydrolase | Metamitron-HPA |
| **Metamitron degradation genes for pathway II (MDGs-II)** | | | | |
| Scaffold 2 | EQB17322.1 | 358 | hydroxylase | Metamitron-DDHM |
| Scaffold 1 | EQB12733.1 | 498 | dehydrogenase | DDHM-DHM |
| Scaffold 2 | ERI43401.1 | 691 | hydrolase | MAHT-MAT |
| Scaffold 5 | ERI40911.1 | 255 | hydrolase | MAHT-MAT |
| Scaffold 11 | ERI41656.1 | 303 | hydrolase | MAHT-MAT |
| Scaffold 13 | ERI40773.1 | 492 | dehydrogenase | DDHM-DHM |
| Scaffold 18 | ERI40787.1 | 396 | dehydrogenase | DDHM-DHM |
| Scaffold 19 | ERI41139.1 | 911 | dehydrogenase | DDHM-DHM |
| Scaffold 20 | KFJ93080.1 | 379 | dehydrogenase | DDHM-DHM |
| Scaffold 21 | ERI40403.1 | 464 | dehydrogenase | DDHM-DHM |
| Scaffold 24 | ERI54260.1 | 252 | dehydrogenase | DDHM-DHM |
| Scaffold 26 | KFJ91335.1 | 399 | dehydrogenase | DDHM-DHM |
| Scaffold 27 | ERI42593.1 | 611 | dehydrogenase | DDHM-DHM |
| Scaffold 28 | ERI42602.1 | 1133 | dehydrogenase | DDHM-DHM |
| Scaffold 30 | ERI41906.1 | 733 | hydrolase | MAHT-MAT |
| HPA: 2-(3-hydrazinyl-2-ethyl)-hydrazono-2-phenylacetic acid;  MBA: methyl benzoylformate acetylhydrazone;  DDHM: 2,3-dihydro-2,3-dihydroxymetamitron;  DHM: 2,3-dihydroxymetamitron;  MAHT: 3-methyl-4-amino-6(2-hydroxy-muconic acid)-1,2,4-triazine-5(4H)-one;  MAT: 3-methyl-4-amino-1,2,4-triazine-5(4H)-one;  BA: benzoylformic acid. | | | | |

**Table S5** Rapid evolutionary gene detection

| Species | Pair gene number | Ka/Ks ratio | | | |
| --- | --- | --- | --- | --- | --- |
|  |  | <0.2 | >1 | >2 | >5 |
| *Rhodococcus* strain MET | 2,990 | 2816 | 2 | 0 | 0 |
| *Rhodococcus* strain IcdP1 | 2,663 | 2501 | 2 | 0 | 0 |
| *Rhodococcus* imtechensis strain RKJ300 | 4,225 | 4,000 | 3 | 0 | 0 |
| *Rhodococcus Jostii* strain RHA1 | 844 | 842 | 0 | 0 | 0 |
| *Rhodococcus erythropolis* strain PR4 | 861 | 859 | 0 | 0 | 0 |
| *Rhodococcus opacus* strain B4 | 4,240 | 4,001 | 4 | 0 | 0 |

**Table S6** Degradation of metamitron at concentrations of 1, 10, and 100 mg/l by the isolate MET in mineral salts medium at pH 7.0 and 30 ºC

| Time  (h) | 1mg/l  (control) | SD | 1 mg/l  (strain) | SD | 10 mg/l  (control) | SD | 10 mg/l  (strain) | SD | 100 mg/l  (control) | SD | 100 mg/l  (strain) | SD |
| --- | --- | --- | --- | --- | --- | --- | --- | --- | --- | --- | --- | --- |
| 0 | 0.877 | 0.004 | 0.852 | 0.022 | 8.98 | 0.05 | 8.81 | 0.14 | 96.62 | 0.95 | 97.16 | 0.41 |
| 2 | 0.875 | 0.020 | 0.235 | 0.024 | 8.96 | 0.17 | 6.49 | 0.11 | 96.60 | 0.95 | 95.22 | 1.69 |
| 4 | 0.870 | 0.019 | 0.018 | 0.008 | 8.91 | 0.18 | 4.74 | 0.40 | 96.50 | 0.95 | 93.60 | 0.43 |
| 6 | 0.865 | 0.018 | 0.002 | 0.0001 | 8.89 | 0.18 | 1.51 | 0.27 | 96.40 | 0.93 | 91.32 | 0.77 |
| 9 | 0.860 | 0.019 | ND | -- | 8.88 | 0.14 | 0.05 | 0.02 | 96.38 | 0.90 | 89.00 | 0.92 |
| 12 | 0.854 | 0.020 | ND | -- | 8.87 | 0.13 | ND | -- | 96.32 | 0.28 | 86.09 | 1.29 |

SD: standard deviation; ND: not detected.

**Table S7** Effect of pH on the degradation of metamitron at the concentration of 10 mg/l by the isolate MET in mineral salts medium

| Time  (h) | pH 5  (control) | SD | pH 5  (strain) | SD | pH 7  (control) | SD | pH 7  (strain) | SD | pH 9  (control) | SD | pH 9  (strain) | SD |
| --- | --- | --- | --- | --- | --- | --- | --- | --- | --- | --- | --- | --- |
| 0 | 9.21 | 0.20 | 9.31 | 0.02 | 9.25 | 0.05 | 9.12 | 0.12 | 8.87 | 0.11 | 8.83 | 0.24 |
| 2 | 9.20 | 0.05 | 9.18 | 0.05 | 9.24 | 0.05 | 6.89 | 0.13 | 5.70 | 0.34 | 4.51 | 0.54 |
| 4 | 9.20 | 0.06 | 9.12 | 0.02 | 9.23 | 0.06 | 5.00 | 0.14 | 3.20 | 0.15 | 3.00 | 0.48 |
| 6 | 9.20 | 0.07 | 8.99 | 0.03 | 9.21 | 0.07 | 1.60 | 0.09 | 2.70 | 0.13 | 2.21 | 0.26 |
| 9 | 9.20 | 0.06 | 8.92 | 0.07 | 9.20 | 0.08 | 0.08 | 0.02 | 2.25 | 0.40 | 1.27 | 0.23 |
| 12 | 9.20 | 0.05 | 8.85 | 0.04 | 9.20 | 0.07 | ND | -- | 1.08 | 0.03 | 1.04 | 0.45 |

SD: standard deviation; ND: not detected.

**Table S8** Effect of temperature on the degradation of metamitron at the concentration of 10 mg/l by the isolate MET in mineral salts medium

| Time (h) | 10ºC (control) | SD | 10ºC (strain) | SD | 20ºC (control) | SD | 20ºC (strain) | SD | 30ºC (control) | SD | 30ºC (strain) | SD | 40ºC  (control) | SD | 40ºC  (strain) | SD |
| --- | --- | --- | --- | --- | --- | --- | --- | --- | --- | --- | --- | --- | --- | --- | --- | --- |
| 0 | 9.41 | 0.20 | 9.31 | 0.13 | 9.42 | 0.09 | 9.31 | 0.06 | 9.09 | 0.09 | 9.01 | 0.08 | 9.33 | 0.22 | 9.11 | 0.03 |
| 2 | 9.40 | 0.54 | 9.23 | 0.07 | 9.41 | 0.50 | 6.79 | 0.03 | 9.00 | 0.40 | 8.62 | 0.14 | 9.25 | 0.50 | 9.01 | 0.03 |
| 4 | 9.40 | 0.64 | 8.86 | 0.07 | 9.40 | 0.50 | 4.52 | 0.31 | 9.00 | 0.40 | 7.11 | 0.31 | 9.25 | 0.48 | 8.83 | 0.08 |
| 6 | 9.40 | 0.55 | 8.48 | 0.02 | 9.40 | 0.50 | 1.90 | 0.29 | 9.00 | 0.45 | 5.51 | 0.04 | 9.25 | 0.48 | 8.73 | 0.03 |
| 9 | 9.40 | 0.32 | 8.02 | 0.17 | 9.40 | 0.50 | 0.36 | 0.29 | 9.00 | 0.49 | 0.47 | 0.24 | 9.25 | 0.46 | 8.45 | 0.05 |
| 12 | 9.35 | 0.13 | 7.55 | 0.11 | 9.39 | 0.11 | ND | -- | 8.94 | 0.09 | ND | -- | 9.22 | 0.08 | 8.37 | 0.02 |

SD: standard deviation; ND: not detected.

**Table S9** Composition of metamitron degradation genes database

| **No.** | **Gene** | **Degradation pathway** | **Enzyme** | **Protein number** |
| --- | --- | --- | --- | --- |
| **Metamitron degradation genes for Pathway I (MDGs-I)** | | | | |
| 1 | *hdl* | metamitron→HPA | hydrolase | 1612 |
| 2 | *dan* | HPA→MBA | deaminase | 153 |
| 3 | *cbe* | MBA→BA | carbonyl enzyme | 23 |
| 4 | *dcl* | BA-benzaldehyde | decarboxylase | 361 |
| 5 | *prc* | benzaldehyde-ring cleavage product | phenyl ring cleavage enzyme | 61 |
| **Total** | | | | **2210** |
| **Metamitron degradation genes for Pathway II (MDGs-II)** | | | | |
| 1 | *hdx* | metamitron→DDHM | hydroxylase | 106 |
| 2 | *dhn* | DDHM→DHM | dehydrogenase | 2058 |
| 3 | *mfe* | DHM→MAHT | meta-fission enzyme | 18 |
| 4 | *hdl* | MAHT→MAT | hydrolase | 1612 |
| 5 | *trc* | MAT→ring cleavage product | triazine ring cleavage enzyme | 15 |
| **Total** | | | | **3809** |
| HPA: 2-(3-hydrazinyl-2-ethyl)-hydrazono-2-phenylacetic acid;  MBA: methyl benzoylformate acetylhydrazone;  DDHM: 2,3-dihydro-2,3-dihydroxymetamitron;  DHM: 2,3-dihydroxymetamitron;  MAHT: 3-methyl-4-amino-6(2-hydroxy-muconic acid)-1,2,4-triazine-5(4H)-one;  MAT: 3-methyl-4-amino-1,2,4-triazine-5(4H)-one;  BA: benzoylformic acid. | | | | |

**Fig. S1**

**Fig. S2**
